# LIMK1 promotes the development of cervical cancer by up-regulating the ROS/Src-FAK/cofilin signaling pathway

**DOI:** 10.18632/aging.206007

**Published:** 2024-07-05

**Authors:** Yajing Jia, Yongping Li, Naiyi Du, Wei Zhao, Yakun Liu

**Affiliations:** 1 Department of Gynecology, The Fourth Hospital of Hebei Medical University, Shijiazhuang, P.R. China

**Keywords:** LIMK1, cervical cancer, oxidative stress, Src

## Abstract

Objective: In this study, we investigated the mechanism of action of LIMK1 in cervical cancer progression.

Methods: The biological role of LIMK1 in regulating the growth, invasion, and metastasis of cervical cancer was studied in SiHa, CaSki cells and nude mice tumor models. The role of LIMK1 in the growth of cervical cancer was evaluated by HE staining. The role of LIMK1 in the invasion, metastasis, and proliferation of cervical cancer was evaluated by cell scratch, Transwell, and monoclonal experiments. The interaction among LIMK1, ROS, and Src was evaluated by Western blotting. The effects of regulating ROS and p-Src expression on LIMK1 in the migration/invasion and proliferation of cervical cancer cells were evaluated through cellular functional assays.

Results: Overexpression of LIMK1 promoted tumor growth in nude mice. Cell scratch, Transwell, and monoclonal experiments suggested that LIMK1 promoted the invasion, metastasis, and proliferation of cervical cancer cells. Western blotting suggested that LIMK1 can promote the expression of ROS-related proteins NOX2, NOX4, p-Src, and downstream proteins p-FAK, p-ROCK1/2, p-Cofilin-1, F-actin and inhibit the expression of p-SHP2 protein. Correction experiments showed that LIMK1 regulated the expression of p-FAK and p-Cofilin-1 proteins by regulating ROS and p-Src. Through the detection of cervical cancer cell functions, it was found that the activation of ROS and p-Src induced by LIMK1 is an early event that promotes the migration, proliferation, and invasion of cervical cancer cells.

Conclusions: LIMK1 promotes the expression of F-actin and promotes the development of cervical cancer by regulating the oxidative stress/Src-mediated p-FAK/p-ROCK1/2/p-Cofilin-1 pathway.

## INTRODUCTION

Cervical cancer is one of the most prevalent and deadly gynecologic malignancies of the female reproductive system, with hundreds of thousands of new cases worldwide each year, and the prognosis is usually poor [1, 2]. In recent years, the incidence of cervical cancer has been increasing year by year, seriously threatening the life and health of women. At present, surgery, radiotherapy, and chemotherapy are mainly used for the treatment of cervical cancer, but these methods are not completely effective [3, 4]. Therefore, it is of great significance to explore the mechanism of the occurrence and development of cervical cancer and find new therapeutic targets.

LIMK1 is a serine protease, which is highly expressed in many tumors and is closely related to the occurrence, development, and metastasis of tumors [5–7]. In this study, we explored the mechanism of action of LIMK1 in cervical cancer progression. At present, there have been some studies on the role of LIMK1 in cervical cancer [8–10]. Studies have shown that the expression level of LIMK1 in cervical cancer tissues is significantly higher than that in normal tissues, and it is closely related to the stage, grade, and prognosis of cervical cancer [10]. Therefore, we speculate that LIMK1 plays an important role in the occurrence and development of cervical cancer. In addition, oxidative stress also plays an important role in the occurrence and development of cervical cancer [11, 12]. Studies have shown that oxidative stress can activate tumor signal transduction, enhance cell survival and proliferation, and drive DNA damage and genetic instability [13]. Studies have shown that the level of reactive oxygen species (ROS) in cervical cancer tissues is significantly higher than that in normal tissues, and the excessive production of ROS is closely related to the progression, metastasis, and prognosis of cervical cancer. At the same time, Src, as a non-receptor tyrosine kinase, is closely associated with ROS [14]. The studies by Takagane et al. showed that the interaction between SHP-2 and Src reduces the activation of SHP-2 [15]. Meanwhile, Jo et al. showed that SHP-2 can inhibit the activity of Src under oxidative stress [16]. FAK-Src promotes the invasion and migration of multiple cancers [17–19]. ROCK and cofilin-1 are the predictive therapeutic targets for the proliferation and metastasis of multiple tumors [20, 21].

Therefore, we speculate that LIMK1 plays an important role in the occurrence and development of cervical cancer by regulating the oxidative stress/Src-mediated signaling pathway. Our study reveals the mechanism of LIMK1 in the progression of cervical cancer and provides new ideas for the treatment of cervical cancer.

## MATERIALS AND METHODS

### Clinical research

### Bioinformatics analysis

Utilized the GEO database (http://www.ncbi.nlm.nih.gov/geo/) on the NCBI (National Center for Biotechnology Information) platform to screen for gene chips, retrieved and downloaded datasets related to cervical cancer. Then, processed the normalization using R language (version 4.1.3) and relevant packages, and conducted differential analysis on the datasets using the Limma R package. Obtained the differential genes DEGs, drawn the volcano plot of differential genes, and the heat map of differential gene clustering. The threshold for differential genes was set to |logFC|>1, p<0.05.

Used the DAVID (Database for Annotation, Visualization, and Integrated Discovery Database) bioinformatics resource database to perform online analysis of DEGs, with *Homo sapiens* genes as the background for Gene Ontology (GO) and Kyoto Encyclopedia of Genes and Genomes (KEGG) enrichment analysis. Identified gene clusters and pathways that have biological feature differences between cervical cancer and normal tissues. Used R language (version 4.1.3) ggplot2 package to draw bar charts for GO up-regulated and down-regulated genes enrichment analysis and scatter plots for KEGG enrichment analysis.

To validate key genes, downloaded cervical cancer and normal tissue datasets and clinical datasets from UCSC Xena (https://xenabrowser.net/), and analyzed them using R language (version 4.1.3). Drawn box plots for disease and normal tissue expression and prognostic survival curves, and plot single-gene expression scatter plots.

### Patient information and sampling

Ten cases of cervical cancer tissues and 10 cases of paracervical cancer tissues surgically removed from June 2021 to June 2023 in the Fourth Hospital of Hebei Medical University were collected. The age of the patients ranged from 32 to 68 years old, with a mean age of (45.71±6.64) years old; all patients were female. Inclusion criteria: ① cervical cancer was diagnosed by biopsy or postoperative pathological examination results; ② no previous systematic anticancer treatment and all of them were the first time to be diagnosed and treated; ③ complete specimens were available and the follow-up data were complete; ④ the patients knew the content of the study and agreed to it. Exclusion criteria: ① Combined with hematologic diseases, thrombotic diseases, systemic lupus erythematosus and other autoimmune diseases; ② Combined with severe hepatic and renal insufficiency; ③ Combined with other malignant tumors; ④ Pregnant and lactating women. The study complied with the ethical requirements of the Declaration of Helsinki and was approved by the Medical Ethics Committee of the Fourth Hospital of Hebei Medical University. All patients gave their informed consent to the study and signed the relevant informed consent form. (Ethics approval number: 2023KS053).

### Immunohistochemical staining

Extract the high-pressure antigen from cancer and pericarcinoma tissue sections using a pH 6.0 sodium citrate solution for 3 min, and then incubate the sections at 30° C for 30 min. After blocking endogenous peroxidase with hydrogen peroxide for 30 minutes, incubate the LIMK1 primary antibody with sections overnight at 4° C. The sections were then washed, incubated with horseradish peroxidase-conjugated secondary antibody for 30 min, washed with PBS, and stained with DAB. After staining, counterstain sections with hematoxylin, dehydrate, and seal with gelatin.

### Animals

### Establishment of animal model

All animal experiments were approved by the Animal Care and Use Committee of the hospital. The study used 32 4-week-old BALB/c nude mice purchased from Henan SKBEX Biology Co., Ltd. Before the experiment began, the animals were reared in the SPF-grade animal house. Cervical cancer cells were infected with lentivirus (Negative Control (NC), LIMK1 overexpression, and LIMK1 knockdown) for 24 hours, and then unilateral subcutaneous axillary injection into nude mice to induce tumors.

### HE staining

The tumor tissues of mice were embedded in paraffin wax and then sliced, soaked in the dewaxing solution for 30 minutes, then soaked in anhydrous alcohol, 90% alcohol, 80% alcohol, and 70% alcohol for 5 minutes, and then sliced by gradient alcohol hydration. After that, the slices were stained with hematoxylin for 4 minutes, washed with running water, and stained. After sealing with gum, the slices were observed under the microscope and photographed.

### Cell culture and transfection

Human cervical cancer cell lines SiHa (Procell, Wuhan, China) and CaSki (Procell) were purchased from the cell bank of the Chinese Academy of Sciences. The cell culture medium consists of Dulbecco's Modified Eagle Medium (DMEM), 1% penicillin-streptomycin, and 10% fetal bovine serum. The cells were cultured in the incubator with 5%CO_2_ at 37° C.

Transfection: The plasmid sequences are as follows. LIMK1 overexpression: 5'-TGC TGA TGG AGT GGA GGT AGG CCA TCG TTT TGG CCA CTG ACT GAC GAT GGC CTC TCC ACT CCA T-3'. NC overexpression: 5'-CCT CCA GTG ACC GCC TAA G-3'. LIMK1 knockdown: 5'-CTC CAG AGG GCT AAG TGT T-3'. NC knockdown: 5'-CUA ACG CAU GCA CAG UCG UAC G-3'. Cervical cancer cells in the logarithmic stage of growth were obtained and re-suspended in serum-free DMEM, cultured with Lipofectamine 3000 (Invitrogen; Thermo Fisher Scientific, Inc.) in the six-well plate, and cultured in the complete medium after 24h [22].

### Transwell migration and invasion experiment

To assess cell invasion, the upper chamber of the Transwell insert was pre-coated with matrix glue at room temperature for 1 hour. The transfected SiHa, CaSki, and NC cells were re-suspended in serum-free DMEM, added to the upper chamber, incubated in the cell incubator for 24 hours, fixed with 4% paraformaldehyde for 30 minutes, stained with 0.1% crystal violet solution at room temperature for 30 minutes, and counted by microscope.

The Transwell migration experiment was conducted as described above, with the exception that no matrix gel was pre-coated on the membrane before seeding the cells.

### Cell scratch experiment

Transfected SiHa and CaSki were inoculated into 24-well plates and incubated in the complete medium for 24 hours to form fused monolayers. Scrape with pipette gun head to form scratches. The culture medium containing 2% serum was used and the experiment was followed up for 24 hours.

### Single-cell cloning

The transfected SiHa and CaSki were prepared into single-cell suspension using DMEM medium, then the cells were inoculated in the 6-well plate for 12 days, and the cells were fixed with 4% paraformaldehyde and stained with 0.1% crystal violet solution to count the visible colonies.

### Western blotting

The transfected cells were lysed using RIPA lysis buffer and the protein concentration was measured with the BCA kit. Protein was isolated by SDS-PAGE (protein loading concentration was 4 μg), the gel was transferred with PVDF membrane, and sealed with 5% skim milk (2 h) at 25° C. The membrane was incubated with LIMK1 (1,000; Abcam, UK), NOX2 (1:1,000; Abcam), NOX4 (1:1,000; Abcam), p-Src (1:1,000; Abcam), p-FAK (1:1,000; Abcam), p-ROCK1/2 (1:1,000; Abcam), p-Cofilin-1 (1:1,000; Abcam), p-SHP-2, GAPDH (1:1,000; Abcam) primary antibodies at 4° C overnight. After washing with TBST, the membrane and the secondary antibody were incubated at room temperature for one hour. Luminescence was done using ECL substrate kit.

### CCK8 assay

10 μl CCK-8 solution (Solarbio, Beijing, China) was added to 96-well plates. Pre-incubate the plates in an incubator for 24 hours (at 37° C, 5% CO_2_). The transfected cervical cancer cells were inoculated into 96-well plates at a density of 2 × 10^3^ cells, and the drugs (5mM NAC/ 1 μM Saracatinib) were added to the plates and incubated for 0 hours. The absorbance at 450 nm was detected using an ELx808 microplate reader (Bio-Tek, USA).

### Statistical analysis

Numerical data are expressed as the mean ± standard deviation (SD) of three independent experiments. Analysis of variance was used for comparison between groups. The difference was statistically significant when the P-value was less than 0.05.

## RESULTS

### Results of bioinformatics analysis

**Figure 1:**
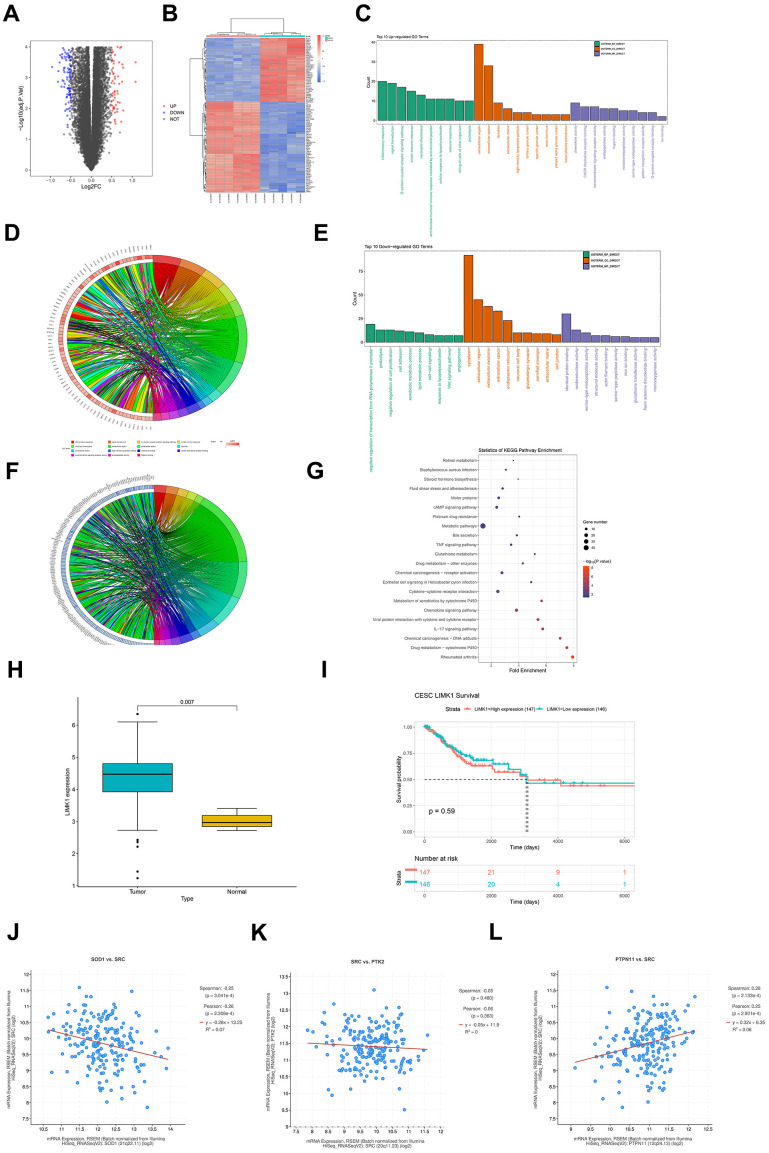
**Raw letter analysis results.** (**A**) Volcano plot of visualized grouped differential genes (DEGs) for dataset GSE173097 was constructed in R software using the ggplot2 package; (**B**) heatmap of cluster analysis of DEGs was plotted using the R package pheatmap; (**C**) biological processes containing the top 10 counts were plotted in the R language environment using GOplot as well as the ggplot2 package, cellular components and molecular functions of up-regulated differentially expressed genes in GO pathway pathway maps; (**D**) enrichment in biological processes such as inflammatory response, signal transduction, G-protein coupled receptor signaling pathway chordal maps; (**E**) GO pathway maps containing differentially expressed genes down-regulated by BP, CC, and MF; (**F**) enrichment in negative regulation of transcription from RNA polymerase II promoter, proteolysis, negative regulation of cell proliferation and other biological processes; (**G**) KEGG enrichment analysis; (**H**) box plot of expression in diseased and normal tissues; (**I**) LIMK1 prognostic overall survival curves; (**J**) LIMK1 and SOD1 plotted as a correlation scatterplot; (**K**) sod1 and SRC plotted as a correlation scatterplot; (**L**) PTPN1 and SRC plotted as a correlation scatter plots.

Searched for cervical cancer-related datasets in the GEO database, finding and downloading the gene expression dataset of cervical cancer-related mRNA, GSE173097, based on the Agilent-045997 Arraystar human lncRNA microarray V3 (Probe Name Version) platform with the probe GPL16956. Quantile normalization of the RNA-seq data of GSE173097 was performed using the R language's limma package. The dataset includes 5 cancerous tissues and 6 normal tissues as analysis samples. Differential gene analysis is conducted on the samples (|logFC|>1, p-value<0.05). The ggplot2 package in R software is used to construct a volcano plot of differentially expressed genes (DEGs) for the dataset GSE173097 (Figure 1A), and the pheatmap package in R software is used to draw the clustering heat map of DEGs (Figure 1B).

Through the DAVID online database tool (https://david.ncifcrf.gov), GO enrichment analysis and KEGG enrichment analysis are performed on the DEGs of dataset GSE173097. The GO analysis integrates GO terms by analyzing the corresponding differentially expressed genes and creates a biological process network of differentially expressed genes. GO categorizes the functional annotations of DEGs into three groups: biological process (BP), cellular component (CC), and molecular function (MF). The GOplot and ggplot2 packages in the R language environment are used to draw the GO pathway diagrams containing the top 10 up-regulated differentially expressed genes in biological processes, cellular components, and molecular functions (Figure 1C), showing enrichment in biological processes such as inflammatory response, signal transduction, G−protein coupled receptor signaling pathway, etc.; chord diagrams (Figure 1D) include GO pathway diagrams of down-regulated DEGs in BP, CC, and MF (Figure 1E), showing enrichment in biological processes such as negative regulation of transcription from RNA polymerase II promoter, proteolysis, negative regulation of cell proliferation, etc.; chord diagrams (Figure 1F). KEGG enrichment analysis indicates enrichment in pathways such as Metabolic pathways, Cytokine−cytokine receptor interaction, cAMP signaling pathway, etc. (Figure 1G).

Downloaded the cervical cancer TCGA dataset CESC from UCSC Xena (https://xenabrowser.net/), which includes datasets of disease and normal tissues, downloaded clinical datasets, and performed analysis using R language (version 4.1.3). Drawn box plots for disease and normal tissue expression (Figure 1H), from which it can be seen that LIMK1 is highly expressed in cancerous tissues (p<0.05). Combined with clinical data, drawn the overall survival (OS) curve of LIMK1 (Figure 1I), from which it can be concluded that LIMK1 has a short survival period.

To explore the co-expression properties of genes in the dataset, the processed dataset was screened, and a scatter plot of the correlation between LIMK1 and SOD1 was drawn (Figure 1J), a scatter plot of the correlation between SOD1 and SRC (Figure 1K), and a scatter plot of the correlation between PTPN1 and SRC (Figure 1L). The scatter plots calculated the Pearson correlation coefficient to assess the correlation between pairs of genes and used the mean line to visually display the average expression level.

### LIMK1 promotes the growth of cervical cancer tumors

**Figure 2:**
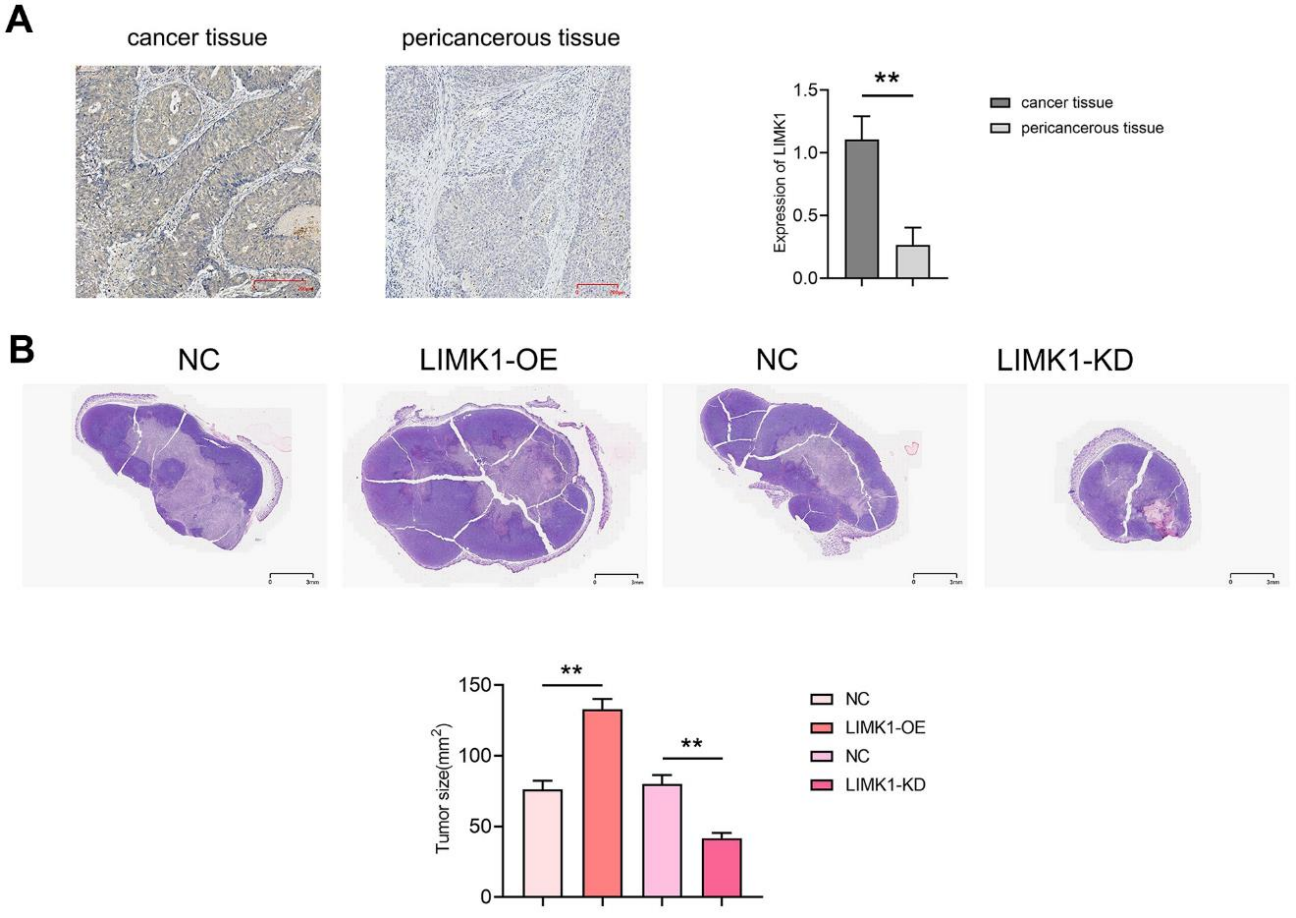
**LIMK1 promoted the growth of cervical cancer.** (**A**) Diagram of LIMK1 immunohistochemical staining results of cancer tissues and paracancerous tissues of cervical cancer patients and statistical results of LIMK1 expression; (**B**) representative micrographs of HE staining results of tumors in each group of mice and statistical histograms of tumor section areas. **P<0.01 indicated statistically significant differences.

First, we used immunohistochemical staining to detect the expression of LIMK1 in cancer and pericancerous tissues of human cervical cancer. The results showed that the relative protein expression of LIMK1 in cancer tissues (1.11±0.19) was significantly higher than that in pericancerous tissues (0.27±0.14). The results of tumor-bearing experiments and HE staining in nude mice showed that compared with the NC group (76.36±5.97), the tumor slice area of the LIMK1-OE group (132.8±7.44) increased significantly. Compared with the NC group (80.03±6.24), the tumor slice area of the LIMK1-KD group (41.56±3.84) was significantly reduced (Figure 2). These results suggest that LIMK1 can promote the occurrence and development of cervical cancer.

### LIMK1 promotes the migration and invasion of cervical cancer cells

**Figure 3:**
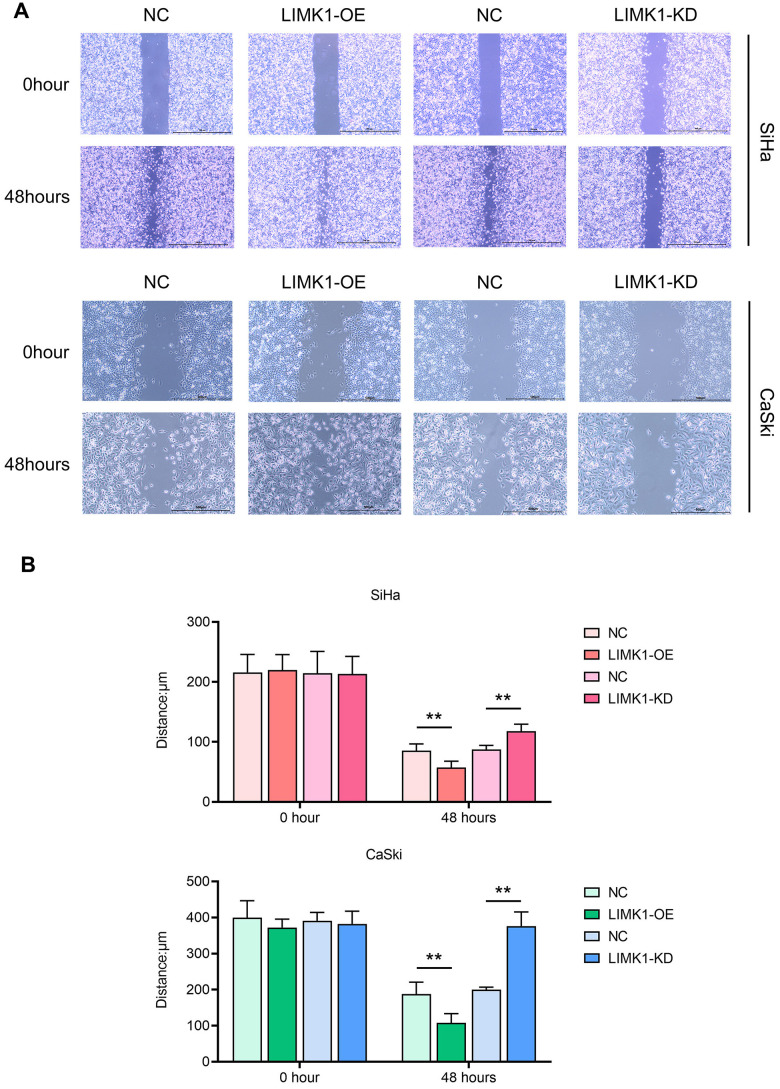
**LIMK1 promoted the migration of cervical cancer cells.** (**A**) Representative images of migration regions at 0 h and 24 h after scratch manufacturing of SiHa and CaSki cells. (**B**) Statistical plots of cell scratch in each group. **P<0.01 indicated statistically significant differences.

**Figure 4:**
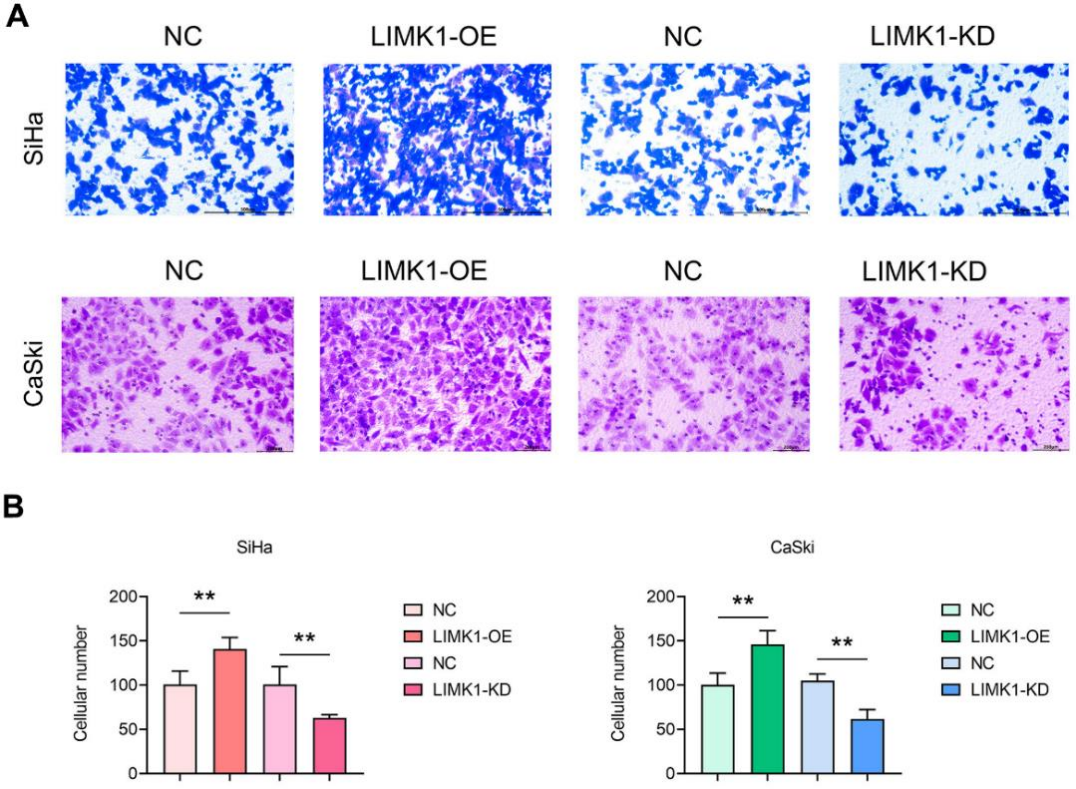
**LIMK1 promoted the invasion of cervical cancer cells.** (**A**) Representative images of SiHa and CaSki cells from Transwell infiltration experiments. (**B**) Statistical graph of the number of SiHa and CaSki cells infiltrated through the basement membrane. **P<0.01 indicated statistically significant differences.

To investigate the effect of LIMK1 on the migration and invasion ability of cervical cancer cells, the results of cell scratch assay showed that at 48 h, the scratch spacing between SiHa cells and CaSki cells in the LIMK1-OE group was significantly lower than that of the NC group, and the spacing between SiHa cells and CaSki cells in the LIMK1-KD group was significantly higher than that of the NC group (Figure 3). The results of Transwell assay showed that the number of migration and invasion of SiHa cells and CaSki cells in the LIMK1-OE group was significantly higher than that of the NC group, and the number of migration and invasion of SiHa cells and CaSki cells in the LIMK1-KD group was significantly lower than that of the NC group (Figure 4). The above data proved that LIMK1 can promote the migration and invasion of cervical cancer cells.

### LIMK1 promotes the proliferation of cervical cancer cells

**Figure 5:**
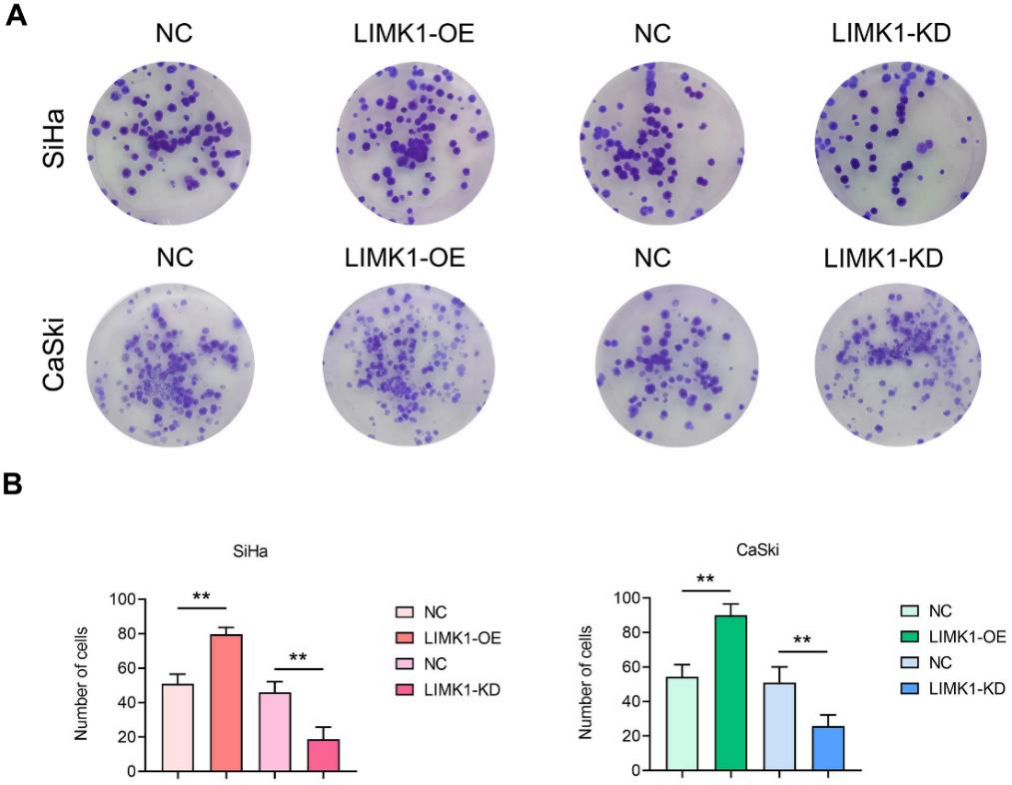
**LIMK1 promoted the proliferation of cervical cancer cells.** (**A**) Representative images of SiHa and CaSki cell monoclonal experiments. (**B**) The results of the clonal formation assay showed that LIMK1 overexpression promoted the formation of cell clones and increased the number of colonies. LIMK1 knockdown inhibited the formation of cell clones, resulting in a decrease in the number of colonies. **P<0.01 indicated statistically significant differences.

To investigate the effect of LIMK1 on the proliferation ability of cervical cancer cells. The results of clone formation experiment showed that the number of clones of SiHa cells and CaSki cells in the LIMK1-OE group was significantly higher than that of the NC group. Compared with the NC group, the number of clones of SiHa cells and CaSki cells in the LIMK1-KD group was significantly reduced (Figure 5).

### LIMK1 regulates the expression of proteins related to the ROS/Src-FAK/cofilin signaling pathway

**Figure 6:**
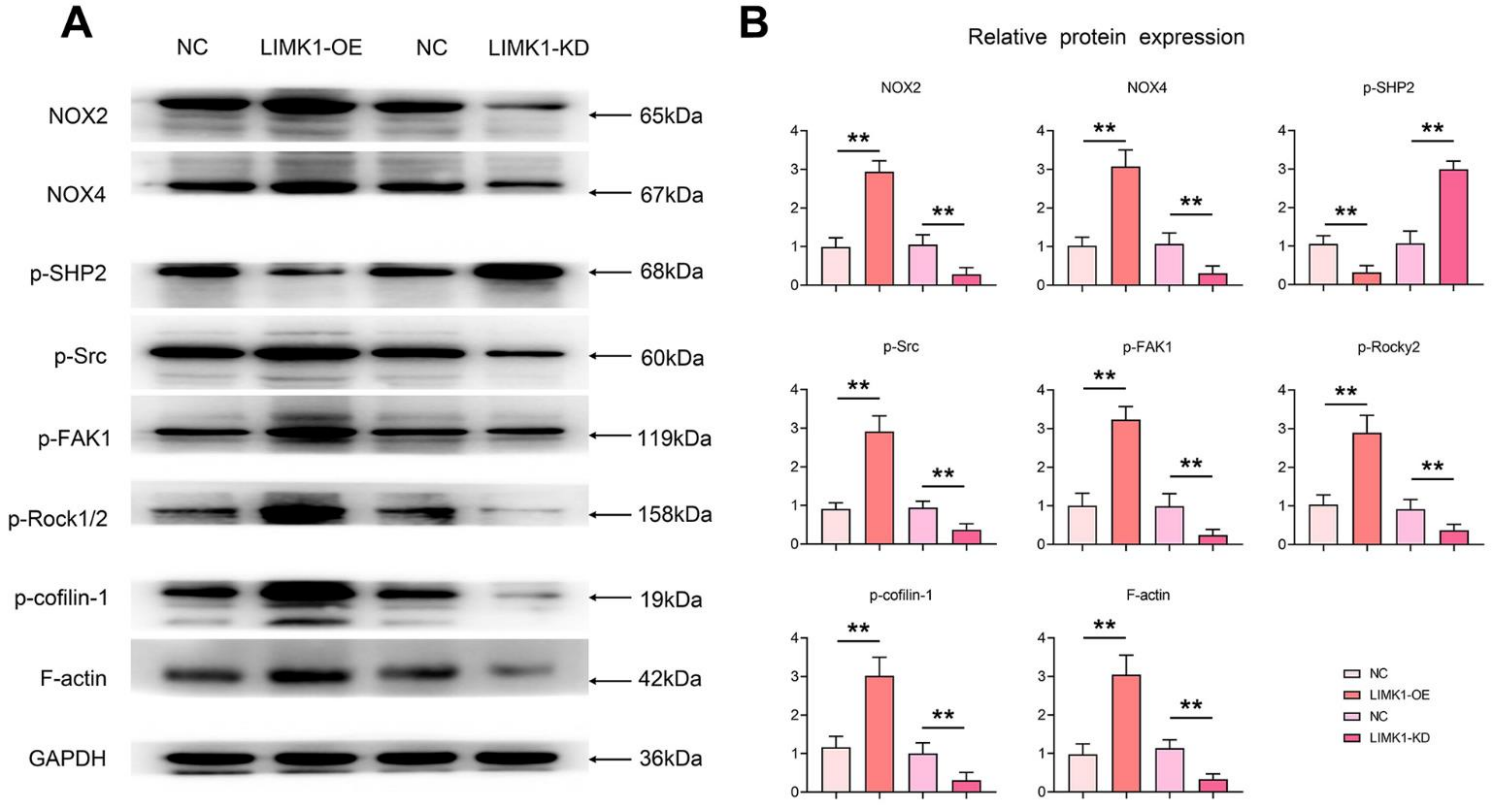
**LIMK1 induced oxidative stress in cervical cancer cells, inhibited p-SHP2 and promoted the expression of p-Src, p-FAK, p-ROCK1/2, and p-Cofilin-1.** (**A**) Effects of LIMK1 on the expression of NOX2, NOX4, p-Src, p-FAK, p-ROCK1/2, p-Cofilin-1, F-actin and p-SHP2 proteins in cervical cancer cells. (**B**) Statistical analysis bar chart of Western blotting. **P<0.01 indicated statistically significant differences.

Western blotting results showed that the relative protein expression of ROS-related proteins NOX2 and NOX4, p-FAK, p-ROCK1/2, p-Cofilin-1, and F-actin was significantly increased, and the expression of p-SHP2 was significantly decreased in the LIMK1-OE group, compared with the NC group. In contrast, relative protein expression of ROS-related proteins NOX2 and NOX4, p-FAK, p-ROCK1/2, p-Cofilin-1, and F-actin was significantly decreased and expression of p-SHP2 was significantly increased in the LIMK1-KD group relative to the NC group (Figure 6).

### LIMK1 mediates the ROS/Src-FAK/cofilin signaling pathway to regulate the proliferation, migration and invasion of cervical cancer cells

**Figure 7:**
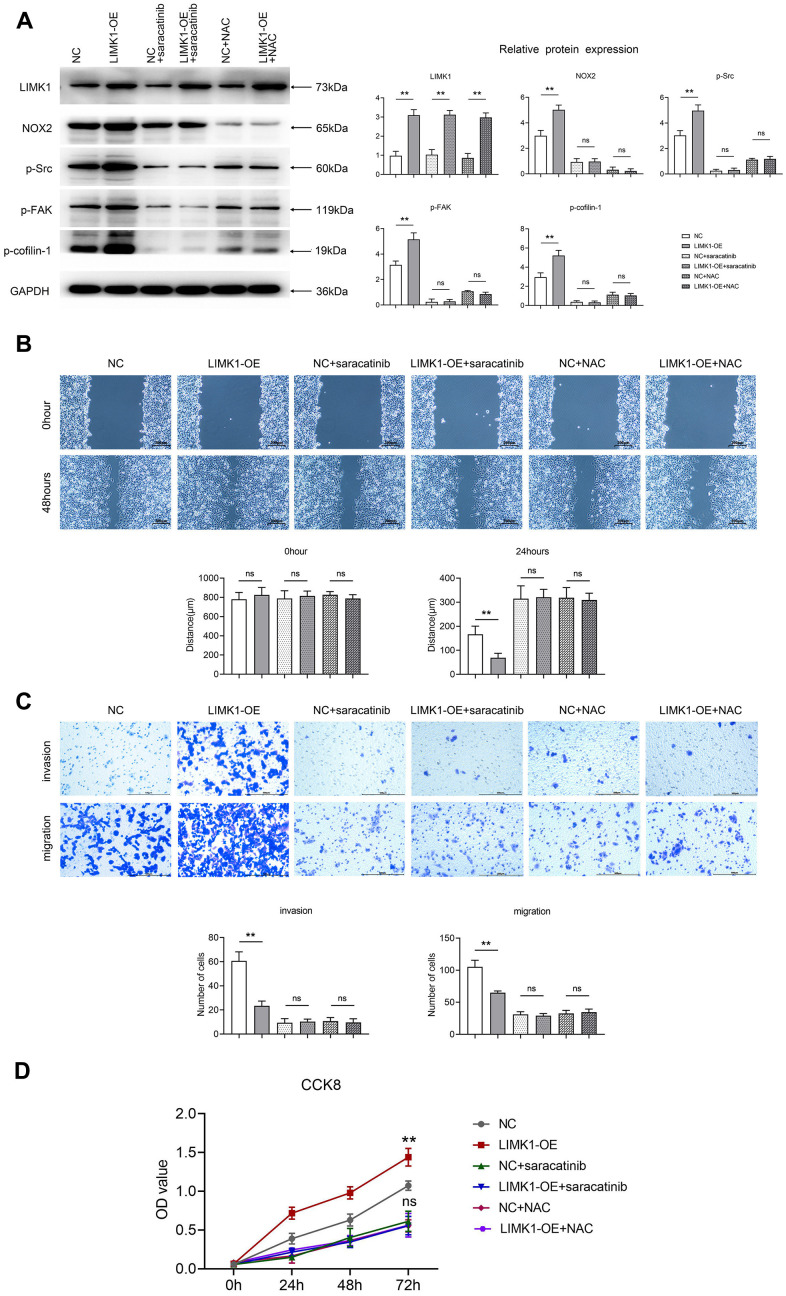
**LIMK1 regulated the expression of p-FAK and p-Cofilin-1 proteins by regulating ROS and p-Src.** (**A**) Effect of 5.0 μM Saracatinib or 5 mM NAC-treated SiHa cells on the expression of LIMK1, NOX2, p-Src, p-FAK, and p-Cofilin-1 proteins in cervical cancer cells and histograms of statistical analyses by Western blotting. (**B**) Plot of the results of the cell scratch experiment and statistics of cell scratch spacing. (**C**) Graphs of the results of Transwell experiments and statistics on the number of cells migrating and invading. (**D**) CCK8 experimental results are plotted. **P<0.01 indicated statistically significant differences; ^ns^P>0.05 indicates that the difference is not statistically significant.

Through the culture of cervical cancer cells by ROS inhibitor NAC and Src inhibitor Saracatinib, we performed correction experiments. Western blotting results showed that the relative protein expression of LIMK1, NOX2, p-Src, p-FAK and p-Cofilin-1 was significantly higher in the LIMK1-OE group relative to the NC group. After the addition of NAC and Saracatinib to the NC and LIMK1-OE groups, respectively, the relative protein expression of NOX2, p-Src, p-FAK and p-Cofilin-1 in the NC and LIMK1-OE groups was significantly reduced and significant differences were eliminated, except for LIMK1 (Figure 7A).

Cell scratch experiment data shows that the overexpression of LIMK1 leads to smaller scratch spacing compared to NC cells. Comparison of scratch spacing between the Saracatinib-NC group and Saracatinib-LIMK1 overexpression group of cervical cancer cells reveals that Saracatinib reverses the effect of LIMK1 on the migration of cervical cancer cells. Similarly, comparison between the NAC-NC group and NAC-LIMK1 overexpression group of cervical cancer cells shows that NAC reverses the effect of LIMK1 on cervical cancer cells (Figure 7B).

The results of the Transwell assay showed that the number of migrating and invading cells was significantly increased in the LIMK1-OE group relative to the NC group. After the addition of NAC and Saracatinib to the NC and LIMK1-OE groups, respectively, the number of migrating and invading cells was significantly reduced and the significant difference was eliminated in both the NC and LIMK1-OE groups, indicating that the activation of LIMK1-induced ROS and p-Src is an early event promoting the invasion of cervical cancer cells (Figure 7C).

**Figure 8:**
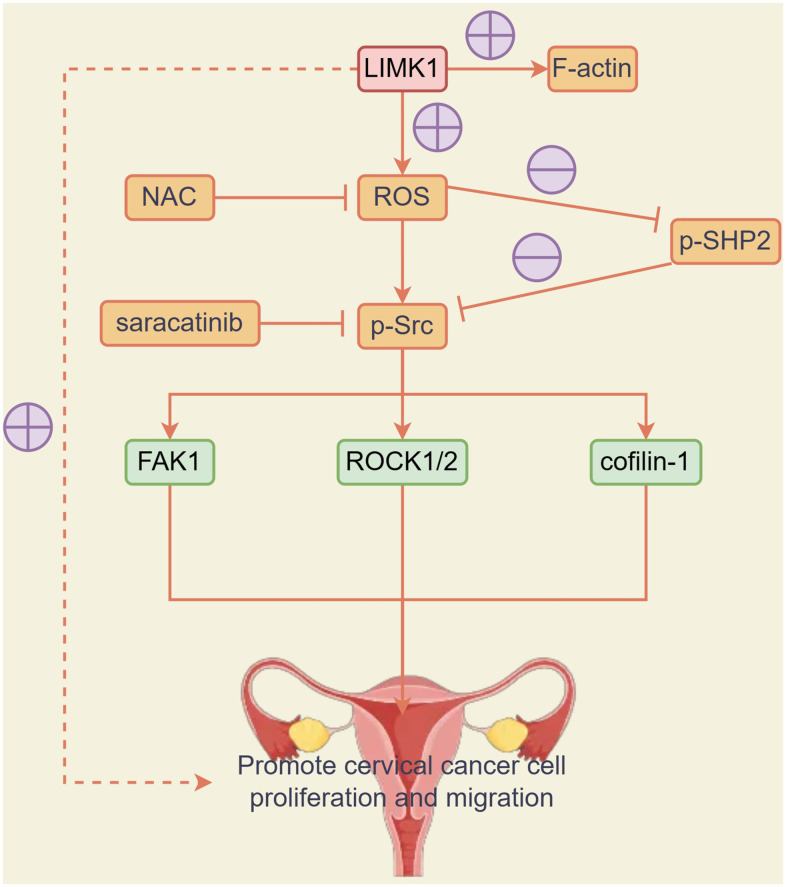
LIMK1 promoted the expression of F-actin and promoted the development of cervical cancer by regulating the oxidative stress/Src-mediated p-FAK/p-ROCK1/2 /p-Cofilin-1 pathway.

The results of CCK8 experiments showed that at 72h, OD values were significantly higher in the LIMK1-OE group relative to the NC group. After the addition of NAC and Saracatinib to the NC and LIMK1-OE groups, respectively, OD values were significantly reduced and significant differences were eliminated in both NC and LIMK1-OE groups, indicating that the activation of LIMK1-induced ROS and p-Src is an early event promoting the proliferation of cervical cancer cells (Figure 7D). In summary, LIMK1 promotes F-actin expression and cervical cancer development by regulating the oxidative stress/src-mediated p-FAK/p-ROCK1/2/p-Cofilin-1 pathway (Figure 8).

## DISCUSSION

Poor prognosis is a common problem in the treatment of cervical cancer. Therefore, it is necessary to study the key molecular mechanisms in the progression of cervical cancer. In this study, we firstly investigated in cancer and paracancerous tissues of cervical cancer patients, and we found that LIMK1 expression was significantly higher in cervical cancer tissues than in paracancerous tissues. It indicated that LIMK1 plays an important role in the progression of cervical cancer. The results suggest that LIMK1 promotes the invasion, metastasis, and proliferation of cervical cancer cells and promotes cervical cancer development by regulating the oxidative stress/SRC-mediated p-FAK/p-ROCK1/2/p-Cofilin-1 pathway. These findings provide new targets and ideas for the treatment of cervical cancer.

In our study, we found that LIMK1 can promote the expression of actin in cervical cancer cells. Actin is an important part of the cytoskeleton, involved in cell movement, pseudopodia formation, and invasion [23]. Our results suggest that LIMK1 can promote the invasion and metastasis of cervical cancer cells by regulating the expression of actin. In addition, we also found that LIMK1 can contribute to the development of cervical cancer by regulating oxidative stress and SRC-mediated signaling pathways. Oxidative stress refers to the disruption of the balance between oxidation and antioxidant in the body, increasing free radicals and reactive oxygen species (ROS). In the development of cervical cancer, the accumulation of ROS can lead to gene mutation, cell proliferation, angiogenesis, and apoptosis inhibition. Our results suggest that LIMK1 can promote ROS production by regulating ROS-related proteins NOX2 and NOX4. Ara Jo et al. confirmed that oxidative stress increased the binding of caveolin-1 and SHP2, inducing SHP2 inactivation [16]. Bertotti's experimental results demonstrated the competitive inhibition relationship of SHP2-Src [24].

In addition, LIMK1 can also contribute to the development of cervical cancer by regulating the Src-mediated signaling pathway. Src is a non-receptor tyrosine kinase involved in a variety of biological processes, including cell proliferation, migration, and invasion. p-FAK and p-ROCK1/2 are classical pathways that promote the invasion and metastasis of cancer cells [25, 26]. Studies have also shown that Cofilin-1 affects the chemical sensitivity of cervical cancer [9]. Cofilin-1 influences the development of a variety of cancer cells. The research of Sousa-Squiavinato et al. suggests that Cofilin-1 may promote actin to regulate actin dynamics, thereby driving membrane processes that cause cancer cells migration and invasion [27]. Our results suggest that LIMK1 can contribute to the development of cervical cancer by regulating the Src-mediated signaling pathway. In conclusion, our study reveals the mechanism of action of LIMK1 in cervical cancer progression, suggesting that LIMK1 can promote the development of cervical cancer by regulating the oxidative stress/Src-mediated p-FAK/p-ROCK1/2/p-Cofilin-1 pathway. These findings provide new targets and ideas for the treatment of cervical cancer.

In the future, the effect of LIMK1 in the treatment of cervical cancer can be verified through genetic analysis and drug therapy trials. For example, small molecule inhibitors targeting LIMK1 could be developed and drug tested in cell and animal models to evaluate their therapeutic efficacy and safety. At the same time, the efficacy of LIMK1 as a therapeutic target can also be verified in clinical trials, providing a new option for the treatment of cervical cancer.

In conclusion, our study reveals the mechanism of LIMK1 in the progression of cervical cancer and provides new targets and ideas for the treatment of cervical cancer. Future studies could further explore the interaction of LIMK1 with other signaling pathways and molecular mechanisms, as well as develop therapies targeting LIMK1, to provide better solutions for the treatment of cervical cancer.
